# Brain Connectivity and Symptom Changes After Transcranial Magnetic Stimulation in Patients With Borderline Personality Disorder

**DOI:** 10.3389/fpsyt.2021.770353

**Published:** 2022-01-18

**Authors:** Tomas Sverak, Pavla Linhartova, Martin Gajdos, Matyas Kuhn, Adela Latalova, Martin Lamos, Libor Ustohal, Tomas Kasparek

**Affiliations:** ^1^Department of Psychiatry, University Hospital Brno and Faculty of Medicine, Masaryk University, Brno, Czechia; ^2^Multimodal and Functional Imaging Laboratory, Central European Institute of Technology, Masaryk University, Brno, Czechia

**Keywords:** transcranial magnetic stimulation, borderline personality disorder, connectivity changes, Go/NoGo task, posterior default mode network

## Abstract

**Objectives:**

Repetitive transcranial magnetic stimulation (rTMS) is an innovative method in the treatment of borderline personality disorder (BPD). We hypothesized that prefrontal rTMS in patients with BPD leads to improved BPD symptoms and that these effects are associated with brain connectivity changes.

**Methods:**

Fourteen patients with BPD received 15 sessions of individually navigated prefrontal rTMS over the right dorsolateral prefrontal cortex. Clinical effects were measured by the Borderline Symptom List 23, UPPS-P, the Difficulties in Emotion Regulation Scale (DERS), the Zung Self-Rating Anxiety Scale (SAS), and the Montgomery and Åsberg Depression Rating Scale (MADRS). Effects of rTMS on brain connectivity were observed with a seed correlation analysis on resting-state fMRI and with a beta series correlation analysis on Go/No Go tasks during fMRI. Assessments were made before and immediately after the treatment.

**Results:**

The assessments after rTMS showed significant reductions in two subscales of UPPS-P, and in DERS, SAS, and MADRS. The brain connectivity analysis revealed significant decreases in amygdala and insula connectivity with nodes of the posterior default mode network (pDMN; precuneus, posterior cingulate cortex, parietal lobules). Connectivity changes were observed both in the resting state and during inhibition. The decrease of amygdala-pDMN connectivity was positively correlated with reduced depression and lack of premeditation after rTMS.

**Conclusions:**

Despite the study limitations (open single-arm study in a small sample), our findings suggest a possible neural mechanism of rTMS effect in BPD, reduced amygdala connectivity with the pDMN network, which was positively associated with symptom reduction.

## Introduction

Borderline personality disorder (BPD) is a serious mental disorder characterized by instability of affect, self-image, and relationships, and by marked impulsivity including self-harm and recurrent suicidal behavior (1). Treatment of BPD is challenging. The primary treatment for BPD is psychotherapy, especially targeted approaches such as dialectical behavior therapy, schema therapy, mentalization-based treatment, and transference-focused therapy (2). Pharmacotherapy is only targeted at symptoms and is not recommended for the treatment of BPD unless targeting specific comorbidities (3, 4). Therapy dropout rates for BPD patients are typically high (5), and the development of new treatment methods for this life-threatening mental disorder is greatly needed.

On the neural level, BPD patients show impairment in multiple networks. Patients with BPD have been reported to show increased involvement of the posterior default mode network (6, 7) and increased connectivity of the anterior cingulate cortex with the amygdala and insula (8). While processing emotional stimuli, patients with BPD show increased and prolonged reactivity of the amygdala, altered prefrontal cortex responses, including the dorsolateral prefrontal cortex (DLPFC), and reduced connectivity between limbic and prefrontal regions as compared to healthy controls in functional magnetic resonance imaging (fMRI) studies (9–12). Reduced gray matter volume was found in BPD patients in the amygdala, insula, and DLPFC (13). Impaired prefrontal-limbic connections are typically described as a mechanism of impaired top-down emotional control in BPD, leading to increased impulsivity and impaired emotion regulation.

Repetitive transcranial magnetic stimulation (TMS, rTMS) is a potentially innovative method in the treatment of BPD. rTMS was used in patients with BPD in nine studies with a total sample of 72 BPD patients (14–22); two of those studies (24 total patients) were placebo-controlled studies (16, 22). Most of the studies targeted the prefrontal cortex because it is hypothesized that high frequency rTMS can increase prefrontal excitability and, subsequently, prefrontal-limbic connectivity. These studies reported good tolerability of the treatment and various effects of rTMS in BPD patients, including improved self-control, emotion regulation, mood, anxiety, and executive functions (23). Only one single case study (15) combined rTMS with neuroimaging, specifically fMRI. Thus, rTMS seems safe and efficient for the treatment of BPD symptoms, but knowledge of the rTMS neural correlate effects is missing and there are no data supporting the hypothesis that prefrontal rTMS leads to increased prefrontal-limbic connectivity.

In this study, patients with BPD underwent high-frequency rTMS of the right DLPFC (rDLPFC). High-frequency rTMS leads to increased cortex excitability, and patients with BPD have been found to have decreased activation predominantly in right DLPFC as compared to healthy controls (11). Also, Swick et al. (24) observed Go/NoGo (GNG) task activation as generally more right sided and DLPFC as connected with attentional executive control or top-down cognitive control and behavioral inhibition. Our goal was to individually target the inhibition network in the DLPFC area (based on the Go/NoGo task) and subsequently strengthen its activity using high-frequency rTMS in patients with BPD.

We hypothesized that prefrontal rTMS in patients with BPD can lead to improved symptoms, specifically to enhanced impulse control and emotion regulation and, based on previous studies, to decreased anxiety and depression. We hypothesized that these effects are associated with brain connectivity changes induced by prefrontal rTMS. We focused on exploring changes in brain connectivity in three regions of interest: the DLPFC, a brain area crucial for emotion and impulse regulation (25, 26) and a stimulation target in this study; the amygdala, a brain area associated with emotion experiencing intensity that is hyperactive in patients with BPD (9); and the insula, a crucial node in the inhibition network (24) and a brain area associated with experiencing negative emotions (27).To maximize the possibility of rTMS enhancing self-control and decreasing impulsivity, we chose individual neuronavigation of the rTMS target based on the fMRI results of a Go/NoGo task. GNG tasks are the most frequent test for measuring waiting impulsivity (28).

## Methods

### Subjects

Fourteen patients with BPD (3 men; age: *M* = 23.57, *SD* = 4.73 years) underwent a prefrontal rTMS protocol. The inclusion criteria were: 5 out of 9 criteria for BPD according to DSM-5 (1), age between 20 and 45, and stable medication for at least 6 weeks before the stimulation and until the end of the stimulation. The exclusion criteria were addiction (except nicotine), bipolar I disorder, and major depressive disorder (current or in the history of the patient), current acute psychotic state, a schizophrenia-spectrum disorder, and contraindications preventing MRI or rTMS.

### Scales and Questionnaires

Self-reported measures were included to assess the clinical effect of the treatment in areas that were hypothesized as possibly improvable by rTMS. Borderline symptoms were measured by the Borderline Symptom List 23 (BSL-23), an established measure for BPD symptom severity (29). Impulsivity was measured by the UPPS-P scale (30, 31), specifically the subscales Lack of Premeditation, Lack of Perseverance, Negative Urgency, and Positive Urgency. Sensation Seeking was left out since it does not seem to be altered in BPD (32). Emotion regulation was measured by the Difficulties in Emotion Regulation Scale [DERS; (33)], and anxiety was measured by the Zung Self-Rating Anxiety Scale [SAS; (34)]. Depression symptoms were measured by the Montgomery and Åsberg Depression Rating Scale [MADRS; (35)] assessed by a clinical psychologist who did not participate in the rTMS patients treatment. The questionnaires were administered to the patients before and after the stimulation protocol. Possible side effects of rTMS were evaluated by research staff before and after each stimulation session, based on the recommendations of McClintock et al. (36).

### Magnetic Resonance Imaging

Functional MRI was done immediately before and after the rTMS protocol with 3T Magnetom Siemens Prisma machine (Siemens Healthcare GmbH, Erlangen, Germany) at the Central European Institute of Technology (CEITEC) in Brno, Czech Republic. The acquisition sequence was three-dimensional magnetization-prepared rapid gradient echo [3D inversion recovery; anatomical T1; 3D MP-RAGE; Repetition time (TR) = 2,300 ms, Echo time (TE) = 2.33 ms, voxel size = 1 × 1 × 1 mm^3^, FoV (224 × 224 mm), Flip Angle (FA) = 8°, 240 sagittal slices]. We used a gradient-echo echoplanar-imaging sequence sensitive to BOLD contrast for acquisition of one task-free and four task fMRI sessions (GNG task) with the following task-free/task parameters: TR = 750/2,280 ms, TE = 35 ms, voxel size = 3 × 3 × 3 mm^3^, FoV (192 × 192 mm), FA = 46°/75°, multiband factor = 4/1, 39/40 transversal slices, 380/153 scans.

### Go/NoGo Task

**Figure 1:**
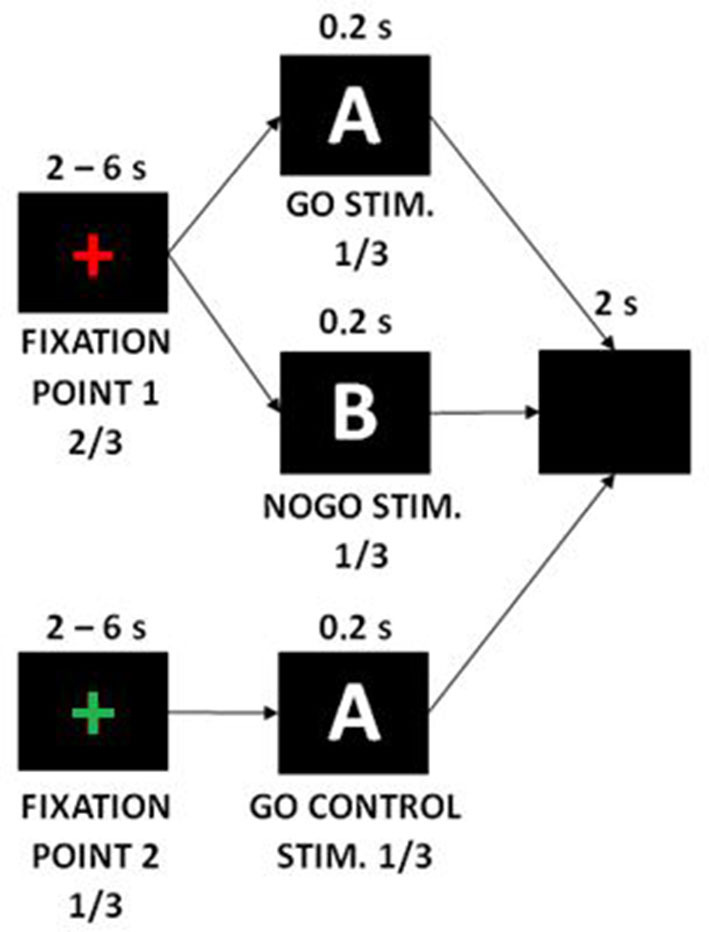
Go/NoGo task design.

We adapted the GNG task according to the task design from Albares et al. (37). In the beginning of each trial, a variable duration fixation cross was presented for 2–6 s, followed by a Go or NoGo stimulus lasting 0.2 s, and finally followed by a post-trial black screen for 2 s. We used white letters A and B on a black background as the Go and NoGo stimuli. The fixation cross was green in 1/3 of cases and red in 2/3 s of cases. The patients were instructed that a Go or NoGo stimulus can appear after the red cross, while only the Go stimulus can appear after the green cross (Go condition, NoGo condition, and Go-control condition; Figure 1). The ratio of Go and NoGo stimuli occurring after the red cross was equal. The patients were further instructed to press a button as quickly as possible when a Go stimulus appeared, but not to press the button when a NoGo stimulus appeared (i.e., to perform inhibition). The task contained 4 blocks of 54 trials each with breaks between the blocks; the whole task lasted ~25 min. NoGo vs. Go contrast was used to analyze the neural correlates of inhibition.

### Individual Neuronavigation

The rDLPFC was targeted individually by stereotactic neuronavigation (software Brainsight, v. 2.02), fMRI data analysis for neuronavigation was performed in SPM12. The rDLPFC anatomical mask was derived from the Destrieux neuroanatomic atlas (38) from FreeSurfer software (39) as a conjunction of the sulcus frontalis inferior, sulcus frontalis medius, and gyrus frontalis medius. Regions of interests were further specified by creating a sphere mask with radius 15 mm around the maximum in rDLPFC derived for the contrast between NoGo and Go conditions and by generating the mask for an area up to 20 mm under the skull. Within the brain area defined by combining those masks, the point of individual peak BOLD signal in the contrast between NoGo and Go conditions was used as rTMS target.

### rTMS

We used the DuoMAG XT magnetic stimulator (Deymed Diagnostic) with a 70BF air-cooled coil. The patients underwent 15 stimulation sessions at 110% of their individual resting motor threshold (RMT) over a period of 3 weeks with one session each working day. One stimulation session contained 1,500 pulses (total 22,500 pulses during the whole procedure) divided into 10 trains with 10 Hz frequency (train interval lasted 10 s, inter-train interval lasted 30 s). The RMT was measured before the first stimulation session and defined according to the Rossini-Rothwell method (40).

### Statistical Analysis

Behavioral and self-reported data analysis was performed in jamovi software v1.6 (41). Differences before and after the rTMS treatment in self-report questionnaires and MADRS scores and in the relative frequency of mistakes after NoGo stimuli in the GNG task (NoGo errors) were analyzed by paired *t*-tests and non-parametric Wilcoxon rank test due to small sample size.

### fMRI Analysis

#### fMRI Data Pre-processing

All functional datasets were preprocessed in SPM12 software (42) running under MATLAB 9.6 (Mathworks Inc., USA) with realign and unwarp, spatial normalization, and spatial smoothing (Gaussian filter with a full width at half-maximum of 6 mm). Task sessions were slice timing corrected before spatial normalization due to longer TR. The data were controlled for spatial abnormalities using the mask_explorer tool (43). We used framewise displacement (FD) criterion (44) to control for excessive movement and excluded all sessions where FD exceeded 0.5 mm in more than 20% of scans (one task-free and two task sessions were excluded).

#### Beta Series Correlation Analysis

We employed beta series correlation analysis (BSCA) (45) to investigate connectivity during distinct stages of the GNG task from selected seeds: left and right amygdala and left and right insula from AAL atlas (46) and rDLPFC from the Human Connectome Project multi-modal parcellation atlas (47). We added 12 movement regressors and white matter (WM) and cerebrospinal fluid (CSF) signals as confounders in the model. In the next step, we correlated the seed beta series (average from the ROI) representing the effects of regressors separately for NoGo and Go trials. To obtain the NoGo vs. Go contrast, we subtracted the connectivity maps for the mentioned conditions. We followed with the paired tests in SPM to identify the connectivity changes before and after rTMS stimulation. Reported group results are based on cluster-level inference at *p* (FWE) <0.05 with an initial cut-off of *p* < 0.001.

#### Seed Correlation Analysis

We used seed correlation analysis (SCA) to analyze connectivity in task-free data with identical seeds as used for BSCA. Before SCA, we filtered low-frequency drifts using high-pass with a cutoff of 1/128 Hz. We also filtered the confounder signals from WM and CSF and the effect of 24 movement regressors. A paired *t*-test was used to assess the stimulation effects. Group results were corrected for multiple testing with cluster-level inference at *p* (FWE) <0.05 with an initial cut-off of *p* < 0.001.

#### Association of Brain Connectivity and Symptom Change

To see whether the observed changes in brain connectivity were associated with changes in the scales measuring BPD symptoms, we performed Spearman correlations between the changes in brain connectivity (T2-T1) and changes in symptom measures (T2-T1; selected measures that showed significant decreases after the rTMS treatment). A positive correlation means that the higher the decrease of brain connectivity, the higher the decrease in measured symptom. A negative correlation means that the higher the decrease of brain connectivity, the lower the decrease in measured symptom. The results were not corrected for multiple comparisons due to the exploratory nature of this analysis and the small sample size.

## Results

RTMS was well-tolerated without any major side effects. Four patients reported headache after the stimulation, usually at the beginning of our protocol, which disappeared spontaneously without any medication after a short time.

### Self-Reported Results

According to the self-reported measures (Table 1), rTMS led to significant reduction in Lack of Premeditation and Lack of Perseverance in the UPPS-P impulsivity scale, improvement in emotion regulation (DERS), reduction of depressive symptoms (MADRS), and borderline significant reduction of anxiety symptoms (SAS). There was no significant change in BSL-23, negative urgency and positive urgency, and no significant change in the percentage of NoGo errors in the GNG task after vs. before the treatment. Parametric and non-parametric tests yielded similar results.

**Table 1:** Self-reported differences before and after rTMS (paired *t*-test and Wilcoxon rank-test).

**Measure**		***N***	***M***	***SD***	***t* (*df*)**	***p***	***d***
					***W***	***p***	***r_***rb***_***
BSL-23	Before	14	43.29	22.28	1.562 (13)	0.142	0.417
	After	14	33.64	19.68	78.000	0.116	0.486
UPPSP-PRE	Before	14	30.29	3.99	**3.095 (13)**	**0.009**	**0.827**
	After	14	27.36	5.64	**91.000**	**0.017**	**0.733**
UPPSP-PER	Before	14	28.79	4.90	**2.692 (13)**	**0.018**	**0.720**
	After	14	25.14	4.80	**79.500**	**0.019**	**0.747**
UPPSP-NU	Before	14	37.14	5.60	1.158 (13)	0.268	0.310
	After	14	34.86	7.33	54.500	0.551	0.198
UPPSP-PU	Before	14	38.14	10.32	1.232 (13)	0.240	0.329
	After	14	35.07	8.09	51.000	0.366	0.308
DERS	Before	14	121.79	20.42	**3.345 (13)**	**0.005**	**0.894**
	After	14	101.29	27.75	**97.500**	**0.005**	**0.857**
SAS	Before	14	48.79	10.33	**2.178 (13)**	**0.048**	**0.582**
	After	14	43.14	11.64	82.500	0.064	0.571
MADRS	Before	14	14.79	5.94	**2.701 (13)**	**0.018**	**0.722**
	After	14	10.86	7.24	**81.000**	**0.014**	**0.780**
NoGo errors %	Before	14	0.12	0.12	0.756 (13)	0.463	0.202
	After	14	0.10	0.09	58.500	0.729	0.114

*BSL-23, Borderline Symptom List 23; UPPSP-PRE, Lack of Premeditation; UPPSP-PER, Lack of Perseverance; UPPSP-NU, Negative Urgency; UPPSP-PU, Positive Urgency; DERS, Difficulties in Emotion Regulation Scale; SAS, Zung Self-Rating Anxiety Scale; MADRS, Montgomery and Åsberg Depression Rating Scale. Bold values meant statistic significant values*.

### fMRI Results

**Figure 2:**
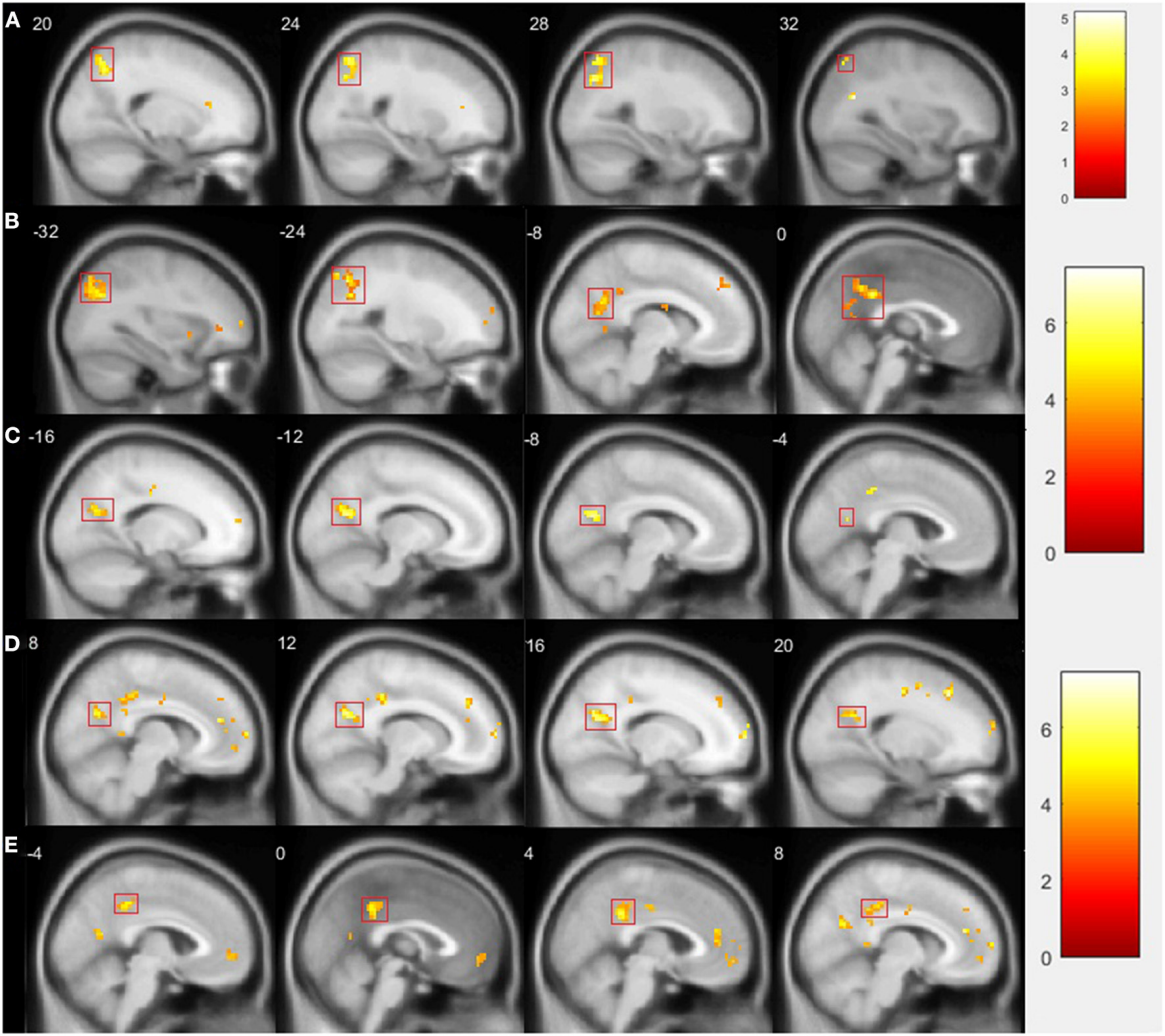
Comparison of fMRI results after vs. before rTMS. Statistically significant changes are marked by a red rectangle (*p* < 0.05 FWE corrected on cluster level). **(A)** Presents decreased connectivity of left amygdala with cluster comprising right precuneus and superior parietal lobule during resting state; **(B)** shows decreased connectivity of left amygdala with cluster comprising left parietal lobule, precuneus, and middle and posterior parts of cingulate gyrus during GNG; **(C)** presents decreased connectivity of left insula with left precuneus during GNG; **(D)** shows decreased connectivity of left insula with right precuneus; **(E)** presents decreased connectivity of left insula with posterior cingulate gyrus.

In the resting state after rTMS vs. before rTMS, we found a decrease in connectivity of the left amygdala with a cluster comprising the right precuneus and superior parietal lobule [peak 27 −70 37 (*t* = 5.00, *p* = 0.025), 78 voxels; Figure 2A]. No significant changes in the right amygdala, insula, or right DLPFC connectivity were observed.

In the GNG task, specifically in the NoGo vs. Go contrast and after vs. before rTMS, we found decreased connectivity of the left amygdala with a cluster comprising the left parietal lobule, precuneus, and middle and posterior parts of cingulate gyrus [peak −15 −67 13 (*t* = 5.99, *p* = 0.000), 374 voxels; Figure 2B]; and a decrease in the connectivity of the left insula with the left precuneus [peak −9 −67 19 (*t* = 5.96, *p* = 0.017), 67 voxels; Figure 2C], with the right precuneus [peak 12 −64 25 (*t* = 5.41, *p* = 0.014), 70 voxels; Figure 2D], and with the posterior cingulate gyrus [peak 6 −40 28 (*t* = 5.11, *p* = 0.010), 74 voxels; Figure 2E]. No significant changes in the right amygdala, right insula, or right DLPFC connectivity were observed.

### Association Between Connectivity and Symptom Change

We observed two trend associations of connectivity change and symptom change (Table 2). Decreased connectivity in the left amygdala with a cluster comprising the right precuneus and right superior parietal lobule in the resting state was positively correlated with decreased depression symptoms (MADRS; *r*_*s*_= 0.52, *p* = 0.067) and Lack of Premeditation (UPPSP-PRE; *r*_*s*_= 0.53, *p* = 0.064). We observed another negative correlation of decreased Lack of Perseverance (UPPSP-PER; *r*_*s*_= –.49, *p* = 0.076) and decreased left insula connectivity with the right precuneus in the inhibition condition.

**Table 2:** Spearman correlations of brain connectivity change with symptom measures change.

**Resting state connectivity change seeds (T2-T1)**		**Scale change**	**Spearman *r* (*p*)**
L amygdala	27 −70 37 (R precuneus, R sup. PL)	DERS	0.41 (0.162)
		MADRS	**0.52 (0.067)**
		SAS	−0.15 (0.635)
		UPPSP-PER	0.06 (0.837)
		UPPSP-PRE	**0.53 (0.064)**
**Go/NoGo Task (NoGo vs. Go contrast) connectivity change**			
L amygdala	−15 −67 13 (L precuneus, mid.-post. CC, L PL)	DERS	−0.27 (0.350)
		MADRS	0.13 (0.657)
		SAS	0.12 (0.691)
		UPPSP-PER	−0.36 (0.213)
		UPPSP-PRE	−0.25 (0.388)
L insula	−9 −67 19 (L precuneus)	DERS	−0.04 (0.887)
		MADRS	0.25 (0.381)
		SAS	−0.13 (0.647)
		UPPSP-PER	−0.13 (0.647)
		UPPSP-PRE	0.23 (0.432)
L insula	12 −64 25 (R precuneus)	DERS	−0.24 (0.401)
		MADRS	0.03 (0.928)
		SAS	−0.30 (0.306)
		UPPSP-PER	**−0.49 (0.076)**
		UPPSP-PRE	0.12 (0.678)
L insula	6 −40 28 (post. CC)	DERS	0.28 (0.326)
		MADRS	0.34 (0.240)
		SAS	0.04 (0.893)
		UPPSP-PER	0.15 (0.620)
		UPPSP-PRE	0.08 (0.780)

*Correlation indicates association between brain connectivity decrease and symptom decrease. R, right; L, left; PL, parietal lobule; CC, cingulate cortex; DERS, Difficulties in Emotion Regulation Scale; MADRS, Montgomery and Åsberg Depression Rating Scale; SAS, Zung Self-Rating Anxiety Scale; UPPSP-PER, Lack of Perseverance; UPPSP-PRE, Lack of Premeditation. Bold values are trend associations of connectivity change*.

## Discussion

This is the first study reporting both behavioral and neural effects of prefrontal rTMS in a sample of patients with BPD. We used high-frequency stimulation of rDLPFC based on the literature about fronto-limbic dysfunction in BPD (i.e., reduced fronto-cingulate activity leading to limbic hyperactivity) which underlies affective dysregulation and impulsivity (9, 11, 48).

### Behavioral Effects

Regarding the performance in inhibition in the GNG task, the number of NoGo errors was low both before and after rTMS, and it did not significantly change. A small number of mistakes is normal in equiprobable tasks such as the one used in our study (28, 49). Regarding the self-reported results, we observed some improvement in all measured variables after the rTMS protocol. The improvement reached statistical significance in emotion regulation (DERS), depression (MADRS), and two UPPS-P impulsivity subscales—Lack of Premeditation and Lack of Perseverance (in other words, the abilities to think before acting and to persist in activities and decisions) and borderline significance in anxiety (SAS). High effects sizes were observed for the changes in Lack of Premeditation and emotion dysregulation, medium effect sizes were observed for changes in Lack of Perseverance and in anxiety and depression levels. The results are consistent with previous studies, specifically the decreased anxiety (19, 22), depression (14, 15, 18–20, 22), impulsivity (15, 19, 20) and affective instability (16). Overall, rTMS treatment had positive effects on some BPD symptoms including anxiety, depression, emotion dysregulation, and impulsivity. Interestingly, only one study (16) used high-frequency stimulation of the right DLPFC such as in our study; other studies used low-frequency stimulation of the same area (19), or high-frequency stimulation of the left DLPFC (14, 15, 19, 20), or DMPFC stimulation (18, 22). Our results are consistent across the previous studies (23). At least two interpretations might be considered here: either the clinical effects are caused by factors other than active stimulation, which might indicate a placebo effect, or the parameters of the stimulation protocol might not play a crucial role in the results, and the rTMS might instead act as a more global prefrontal intervention (thanks to cortico-cortical connections).

### Neural Effects

In both the resting state and inhibition conditions, we found decreased left amygdala connectivity with the precuneus (PCN), posterior cingulate cortex (PCC), and parietal lobules (PL) after rTMS. These regions are parts of the posterior default mode network (pDMN), which is associated with self-related (personal) and future-oriented reflection and thinking and emotional and attributional evaluation of situations (50, 51).

Previous studies reported increased involvement of pDMN in patients with BPD as compared to healthy controls, suggesting higher self-referential thinking (6, 7). Increased connectivity of the amygdala with pDMN areas has been found to be associated with a number of negatively experienced phenomena, such as depressive rumination [PCC (52)], sleep deprivation [PCC, PCN (53)], complicated grief [PCN (54)], and also history of abuse [PCC, PCN (55)] or risk-taking [PCC, PCN (56)]. Increased connectivity of the amygdala with PCN has been also identified in adolescents who attempted suicide or had high suicidal ideation as compared to adolescents with low suicidal ideation and healthy controls (57).

Importantly, decreased amygdala connectivity with pDMN (in resting state only) was positively correlated with decreased depressive symptoms and with decreased lack of thinking before acting and planning (Lack of Premeditation). This means that the larger the decrease in amygdala-pDMN connectivity in the resting state after rTMS as compared to before rTMS, the greater the improvement in depressive symptoms and premeditation ability. Thus, our results are consistent with previous literature reporting positive associations of amygdala-pDMN connectivity and borderline or depressive symptoms. Decreasing amygdala-pDMN connectivity seems to be beneficial in patients with BPD and was achieved after the prefrontal rTMS protocol in our study.

In the inhibition condition, we found decreased connectivity of the PCN and PCC with the left insula after rTMS as compared to before rTMS. Again, there was a decrease of pDMN areas connectivity. The insula processes interoceptive information to engage externally oriented attention and internal cognitive control, helps with integrating information from the limbic system, and is a major part of the inhibition network (24, 58, 59). The PCC, PCN, and insula have been found to be associated with an increased sense of personal agency, with the insula correlating specifically with personal agency in negative situations (50). Higher interconnectedness in the PCN and insula has been found in people with a history of childhood maltreatment (60). In another study, increased connectivity of the insula and PCN distinguished posttraumatic stress disorder patients with and without dissociation symptoms (61). The pDMN has been found to be deactivated during pain, while the insula is activated by pain (62). Increased pDMN-insula connectivity has been reported in patients with BPD during pain processing (63); while decreased pDMN-insula connectivity has been observed in healthy people (64), which might reflect a disturbance in pain processing in BPD and suggest more self-referential experiencing of pain in BPD (63).

In our study, we found a negative correlation of decreased insula-pDMN connectivity in the inhibition condition and symptom reduction—specifically with reduced Lack of Perseverance. Though previous studies suggest that decreasing insula-pDMN connectivity could be beneficial in patients with BPD, our data did not support the hypothesis that reduction in this connectivity is positively associated with symptom changes in patients with BPD. Moreover, it should be noted that we found the decrease of pDMN-insula connectivity only in inhibition condition and not in resting state. Thus, the effect might be more situation specific or task related than general, while previous studies usually report increased pDMN-insula connectivity in resting state or during pain processing.

Overall, previous studies suggest that increased connectivity of pDMN with the amygdala and insula is associated with increased self-referential and self-attributional thinking, especially in negatively perceived situations. These connectivity increases are then associated with symptoms commonly associated with BPD, such as depressive mood, risk-taking, and suicidality with pDMN-amygdala connectivity, and dissociation and disturbed pain processing with pDMN-insula connectivity. Increased connectivity in both cases have been linked to adverse experiences in childhood. Our study reported decreased pDMN-amygdala and pDMN-insula connectivity and decreased BPD symptoms. We observed decreased pDMN-amygdala connectivity both in the resting state and during an inhibition task. This result suggests that negative self-referential thinking was decreased during resting and during a task activity focused on impulse control. We found that symptom reduction was positively correlated with decreased amygdala-pDMN connectivity, but negatively correlated with insula-pDMN connectivity. Thus, the reduction of negative self-referential thinking associated with reduced pDMN connectivity with the amygdala seems to be a beneficial effect for patients with BPD and a candidate mechanism of the rTMS effect in patients with BPD.

In contrast to our initial hypothesis, we did not find any changes in the connectivity of the dorsolateral prefrontal area stimulated by rTMS. Thus, the mechanism of how prefrontal stimulation led to the connectivity and symptom changes observed in this study remains unclear. However, changes in DMN connectivity after high-frequency prefrontal stimulation were found in previous studies in healthy volunteers and in patients with depression and PTSD (65–67). Another study examining the neural effects of high-frequency DLPFC stimulation in 60 healthy people found no changes in DMN connectivity, but multiple increases in the connectivity of the cingulate and frontoparietal areas (68); this was not observed in our study. Several possible interpretations might be drawn concerning how prefrontal stimulation can induce distant connectivity changes without influencing the stimulation area connectivity *per se*. First, it is possible that the effects are induced by factors other than the intervention, including a placebo effect. Double-blind sham-controlled studies are needed to shed more light on this possibility. Second, prefrontal rTMS might actually induce distant beneficial brain changes, which would correspond with the hypothesis that the precise stimulation site might not be a crucial parameter at all. Third, it is possible that the current study did not have sufficient power to show prefrontal connectivity changes, and replication on a higher sample is needed. Moreover, medication in patients with BPD might influence the results since limbic hyperactivity has been found to be moderated by medication status in BPD patients (11). Overall, no existing data currently support the hypothesis that prefrontal rTMS leads to symptom reduction by increasing the prefrontal-limbic connectivity, but symptom reduction might be achieved by prefrontal rTMS through distant brain connectivity changes, such as the decrease of pDMN-amygdala connectivity observed in this study.

### Limitations

The major limitation of our study is the small sample size and absence of a control group. The placebo effect is usually relatively high in rTMS studies (69), and there is a risk that this study was influenced as discussed above. On the other hand, this is the first study to report the neural effects of rTMS in BPD patients. Our results should be taken as exploratory and hypotheses-generating; they should be verified in a placebo-controlled study.

Another possible limit is the choice of behavioral paradigm for individual neuronavigation. We chose this task to identify a DLPFC target most strongly related to impulse control within the aim to reduce impulsivity. However, patients with BPD typically show impulsive behavior under the influence of emotions (32, 70, 71). A different task activating inhibition under emotions or emotion regulation could be more appropriate for targeting rTMS in patients with BPD. On the other hand, given the existing studies of rTMS in BPD, the rTMS target does not seem to play any crucial role in achieving symptom improvement. We only observed changes in connectivity from the right DLPFC, but we did not analyze changes in connectivity within the whole prefrontal cortex. This could be also the reason we observed only distant changes in brain connectivity.

## Conclusion

This study reports on behavioral and neural effects of high-frequency prefrontal rTMS in patients with BPD. After the stimulation protocol, we observed reduced emotion dysregulation, impulsivity, anxiety, and depressive symptoms. On the neural level, we identified decreased connectivity of the posterior default mode network areas with the amygdala and insula; this might reflect decreased negative self-referential thinking. Reduced amygdala-pDMN connectivity is a candidate mechanism of an rTMS effect in patients with BPD. Sham-controlled studies examining the effect of brain target and stimulation parameters for rTMS are needed.

## Data Availability Statement

The data that support the findings of this study are available from the corresponding author, Tomas Sverak, upon reasonable request.

## Ethics Statement

The studies involving human participants were reviewed and approved by Ethics Commission of University Hospital Brno Jihlavská 20, CZ - 625 00 BRNO. The patients/participants provided their written informed consent to participate in this study.

## Author Contributions

All authors listed have made a substantial, direct, and intellectual contribution to the work and approved it for publication.

## Funding

This work was supported by the Ministry of Health of the Czech Republic, Grant No. NU20-04-00410; by the Ministry of Education, Youth and Sports of the Czech Republic, specific university research Project No. MUNI/A/1664/2020; and by the Ministry of Health of the Czech Republic, conceptual development of a research organization (University Hospital Brno, FNBr, 65269705).

## Conflict of Interest

The authors declare that the research was conducted in the absence of any commercial or financial relationships that could be construed as a potential conflict of interest.

## Publisher's Note

All claims expressed in this article are solely those of the authors and do not necessarily represent those of their affiliated organizations, or those of the publisher, the editors and the reviewers. Any product that may be evaluated in this article, or claim that may be made by its manufacturer, is not guaranteed or endorsed by the publisher.
